# Comparison of neutrophil:lymphocyte ratios following coronary artery bypass surgery with or without cardiopulmonary bypass

**DOI:** 10.5830/CVJA-2015-015

**Published:** 2015

**Authors:** Mustafa Aldemir, Fahri Adalı, Görkem Çarşanba, Evren Tecer, Elif Doğan Bakı, Hanife Uzel Taş

**Affiliations:** Department of Cardiovascular Surgery, Faculty of Medicine, Afyon Kocatepe University, Turkey; Department of Cardiovascular Surgery, Faculty of Medicine, Afyon Kocatepe University, Turkey; Department of Cardiovascular Surgery, Faculty of Medicine, Afyon Kocatepe University, Turkey; Department of Cardiovascular Surgery, Faculty of Medicine, Afyon Kocatepe University, Turkey; Department of Anaesthesiology, Faculty of Medicine, Afyon Kocatepe University, Turkey; Department of Public Health, Faculty of Medicine, Afyon Kocatepe University, Turkey

**Keywords:** myocardial revascularisation, cardiopulmonary bypass, off-pump technique, neutrophil:lymphocyte ratio

## Abstract

**Objective:**

Coronary artery bypass graft (CABG) surgery may induce postoperative systemic changes in leukocyte counts, including leukocytosis, neutrophilia or lymphopenia. This retrospective clinical study investigated whether offpump coronary artery bypass (OPCAB) surgery working on the beating heart without extracorporeal circulation could favourably affect leukocyte counts, including neutrophil-tolymphocyte (N:L) ratio, after CABG.

**Methods:**

In this study, 30 patients who underwent isolated CABG with cardiopulmonary bypass (CPB), and another 30 patients who underwent the same operation without CPB between May 2010 and May 2013, were screened from the computerised database of our hospital. Pre-operative, and first and fifth postoperative day differential counts of leukocytes with the N:L ratio of peripheral blood were obtained.

**Results:**

A significant increase in total leukocyte and neutrophil counts and N:L ratio, and a decrease in lymphocyte counts were observed at all time points after surgery in both groups. N:L ratio was significantly higher in the CPB group compared with the OPCAB group on the first postoperative day (20.73 ± 13.85 vs 10.19 ± 4.55, *p* < 0.001), but this difference disappeared on the fifth postoperative day.

**Conclusion:**

CPB results in transient but significant changes in leukocyte counts in the peripheral blood stream in terms of N:L ratio compared with the off-pump technique of CABG.

## Objective

Coronary artery bypass grafting (CABG) is the most common procedure in cardiovascular surgery. However the procedure itself is associated with significant morbidity and mortality rates.

It is well known that large changes in immune reactivity occur during or after cardiac surgical operations.^1^ Surgical trauma has a well-known effect on increased immune mediator levels.^2^ Production of reactive oxygen species, decreased barrier function, induction of hypoperfusion, and tissue destruction are examples of adverse outcomes resulting from severe activation of the native immunity.^3^ Cardiopulmonary bypass circuit devices play a key role at that point, with contact activation of both cellular and humoral components of the blood accepted as major liability issues. T and B cells of the adaptive immune system are affected mostly in the early postoperative period with some delay in the course of surgery.^4^

In open-heart surgery, risk stratification has mostly been done using the European System for Cardiac Operative Risk Evaluation (EuroSCORE).^5^ However there are some concerns about overestimation/underestimation with the EuroSCORE, which is why more reliable predictors are needed.

There are many studies in the literature about the relationship between inflammation and adverse cardiovascular outcomes.^6^ In this era, some biomarkers of inflammation have been investigated, such as total white blood cell count (WCC), a predictor of mortality after coronary artery bypass grafting.^7^ However subtypes of WCC or ratios between them have been shown to be more valuable in the prediction of outcomes.^8^ One of these is the neutrophil:lymphocyte ratio, a potentially useful biopredictor of inflammation in cardiovascular disease.^9^ It is inexpensive, readily available and easily calculable.

In screening the literature, there are some studies on its prognostic value after cardiac operations,^10^ but there are no published studies on the relationship between cardiopulmonary bypass and the neutrophil:lymphocyte (N:L) ratio. Therefore, the current study was conducted to investigate the N:L ratio as a measure of systemic inflammation and its relationship, if any, with cardiopulmonary bypass.

## Methods

This retrospective clinical study was performed on 60 patients who underwent isolated CABG surgery at our institution, Department of Cardiovascular Surgery, Kocatepe University, Afyonkarahisar, Turkey, between May 2010 and May 2013. This clinical retrospective study was approved by the local ethics committee of the Faculty of Medicine, Afyon Kocatepe University.

All patients had coronary artery disease with varying degrees of stenosis of the left anterior descending coronary artery. Patients with left main or left main equivalent coronary artery disease were also included in the study. The data of the 60 cases were collected retrospectively from a computerised clinical database. We selectively collected the data for 30 patients (group I, *n* = 30) who were operated on using the on-pump technique (with cardiopulmonary bypass) and another 30 patients (group II, *n* = 30) who were operated on using the off-pump technique (without cardiopulmonary bypass).

Exclusion criteria of the study were as follows: CABG surgery associated with valvular replacement or any other procedure, circulatory support with intra-aortic balloon pump before surgery, pre-operative ejection fraction less than 30%, recent myocardial infarction (less than three months), emergency operation, re-operation, pre- or postoperative infection (obtained from progress notes in the patient files), immunological disease, tumour, acute or chronic renal failure [pre-operative renal insufficiency was defined as a serum creatinine level ≥ 1.5 mg/dl (132.6 μmol/l) prior to CABG], respiratory impairment, prior stroke, peripheral vascular disease and coagulopathy. Patients with postoperative pulmonary, infectious, neurological or gastrointestinal complications, re-exploration for bleeding, or cardiac tamponade were also excluded from the study.

Relevant demographic and peri-operative clinical data were collected for the 60 patients using the above database, and findings in the two groups (on- and off-pump) were compared. The specific pre- and intra-operative data obtained for each case were patient age and gender, history of hypertension, defined as systolic blood pressure > 160 mmHg or diastolic blood pressure > 100 mmHg, diabetes defined as fasting blood glucose levels > 140 mg/dl (7.77 mmol/l) and the use of oral anti-diabetic medication or insulin dependency, smoking, unstable angina pectoris, prior percutaneous transluminal coronary angioplasty, left ventricular ejection fraction, presence or absence of left main coronary artery disease, and number of grafts per operation. The postoperative data collected were mechanical ventilation time, need for inotropic or intra-aortic balloon pump support, peri-operative myocardial infarction (immediate postoperative period), dysrythmias, length of stay in the intensive care unit (ICU) and overall hospital stay.

Pre-operative, and first and fifth postoperative day total leukocyte counts and differential counts (neutrophils and lymphocytes) were taken from patient files in our hospital archives. The calculation of N:L ratios was entrusted to one of our authors who was blinded to the neutrophil and lymphocyte samples from the two groups.

## Surgical procedure

All patients were given the same anaesthesia protocol. They were pre-medicated with midazolam (0.05 mg/kg IV). On the operating table, cannulae were inserted in a peripheral vein, the radial artery and the right jugular vein. Standard monitoring included pulse oximetry, leads II and V5 of the ECG for heart rate and automated ST-segment trend analysis, continuous measurements of arterial and central venous pressures, nasopharyngeal temperature, and end-tidal capnography.

A balanced anaesthetic technique included fentanyl (bolus of 1–2 mg/kg followed by an intermittent bolus of 1–2 mg/kg/h), etomidate (bolus of 0.2—0.3 mg/kg), esmeron (bolus of 1 mg/kg and an intermitant bolus of 0.3 mg/kg/h) and inhaled sevofluorane (2–3% in the pre-bypass period and 1–1.5% in the bypass period). Ventilation was modified in each patient to reach partial arterial oxygen pressure above 150 mmHg and partial arterial carbon dioxide pressure above 45 mmHg. A conventional median sternotomy was performed in all patients.

In group I (on-pump), cardiopulmonary bypass (CPB) was established in a standardised manner with the use of a roller pump and non-pulsatile flow (2.4 l/m^2^/min). A heparinisation protocol of 300 U/kg was followed to maintain clotting time at longer than 400 s. Patients were cooled to 32°C when distal anastomosis was being performed, and were warmed to 36°C before weaning from CPB.

After aortic cross-clamping, cold-blood cardioplegia was accomplished with anterograde delivery through the aortic root for initial diastolic arrest of the heart, and intermittently after each distal anastomosis. A final dose of ‘hot-shot’ cardioplegia was also administered antegradely just before the aorta was unclamped. Protamine was used to reverse the effects of heparinisation.

In group II (off-pump), by adjusting the operating room temperature, hypothermia was avoided. Partial anticoagulation was accomplished with 1–2 mg/kg body weight of heparin until a target activated clotting time (ACT) of 250 s was achieved. Octopus 4 (Medtronic Inc, Minneapolis, MN, USA) was used as cardiac stabiliser.

In order to obtain a bloodless anastomotic field after arteriotomy, we did the following: after opening the distal artery, intracoronary shunts (Clearview intracoronary shunt, Medtronic Inc, USA) were inserted into the coronary artery for each anastomosis, which were 1.5, 2.0 or 2.5 mm in size according to the coronary artery lumens, and shunts were removed after the last suture just before tying. Heparin was not neutralised by protamine sulfate at the end of the operation.

## Statistical analysis

The values obtained from the groups were compared to evaluate N:L ratios after coronary artery surgery with or without cardiopulmonary bypass. Statistical analysis was performed with SPSS version 15.0 (SPSS, Inc, Chicago, IL) software. Compliance of variables with a normal distribution was analysed with visual (histogram and probability plots) and analytical methods (the Kolmogorov–Smirnov test). Descriptive analyses were provided as mean and standard deviation.

Relationships between cardiopulmonary bypass and N:L ratios and pre-, intra- and postoperative factors were analysed using different methods: the independent samples t-test for normally distributed continuous variables (expressed as mean ± SD), and chi-square and Fisher’s exact tests for categorical variables, as appropriate. The results were assessed within a 95% reliance and at a significance level of *p* < 0.05.

## Results

This study comprised 60 patients who underwent CABG surgery. Thirty of the patients were operated on with CPB (group I) and the other 30 without bypass (group II). The number of bypassed grafts were different between the two groups, being statistically higher in group I than in group II (3.20 ± 0.88 and 1.93 ± 0.78, respectively, *p* < 0.001). Hospital stay was longer in group I (8.97 ± 2.39 days) than in group II (5.90 ± 2.07 days), and this difference was also statistically significant (*p* < 0.001). Except for the difference in number of grafts and hospital stay, patient demographic and peri-operative characteristics were similar in the two groups (Table 1).

**Table 1:** Demographic and peri-operative clinical data of the patients

	*Group I (n = 30)*	*Group II (n = 30)*	*p-value*
Males, *n* (%)	20 (66.7)	15 (50.0)	0.190
Age (years)	63.73 ± 10.49	68.10 ± 6.98	0.102
DM, *n* (%)	14 (46.7)	12 (40.0)	0.602
Smoking, *n* (%)	16 (53.3)	14 (46.7)	0.606
Hypertension, *n* (%)	11 (36.7)	17 (56.7)	0.121
USAP, *n* (%)	2 (6.7)	4 (13.3)	0.671
Previous PTCA, *n* (%)	3 (10.0)	3 (10.0) 9 (30.0)	3 0.053
Pre-operative EF (%)	49.73 ± 10.45	49.63 ± 12.60	0.982
LMCA disease, *n* (%)	2 (6.7)	0 (0.0)	0.492
No of grafts	3.20 ± 0.88	1.93 ± 0.78	< 0.001
Postoperative inotropic need, *n* (%)	12 (40.0)	11 (36.7)	0.791
Postoperative IABCP need, *n* (%)	6 (20.0)	1 (3.3)	0.103
Postoperative MI, *n* (%)	2 (6.7)	0 (0.0)	0.492
Postoperative dysrhythmia, *n* (%)	8 (26.7)	11 (36.7)	0.405
Mechanichal ventilation time (hrs)	5.73 ± 2.70	5.30 ± 1.53	0.897
ICU stay (days)	1.87 ± 0.68	1.83 ± 1.20	0.256
Hospital stay (days)	8.97 ± 2.39	5.90 ± 2.07	< 0.001

Values are mean (± standard deviation) or median (range) as appropriate. Group I: group with cardiopumonary bypass, group II: group with offpump coronary artery bypass, DM: diabetes mellitus, USAP: unstable angina pectoris, IABCP: intra-aortic balloon counterpulsation, ICU: intensive care unit, PTCA: percutaneous transluminal coronary angioplasty, EF: ejection fraction, LMCA: left main coronary artery, MI: myocardial infarction.

For pre-operative values of the leukocyte screen, the two groups were similar in terms of leukocyte, neutrophil and lymphocyte counts and N:L ratios (Table 2). However, comparing systemic leukocyte counts affected by the surgical stress of CABG alone, with pre-operative values, a significant increase in counts of total leukocytes and neutrophils, and N:L ratio, and a decrease in lymphocyte counts was found on the first and fifth days after surgery, regardless of the technique used (with or without CPB) (Table 2).

**Table 2:** Comparison of values at all time points between the two groups

	*Group I (n = 30)*	*Group II (n = 30)*	*p-value*
Pre-operative			
Total leucocytes (10^3^/ml)	7.59 ± 1.78	7.07 ± 1.55	0.403
Neutrophils (10^3^/ml)	4.19 ± 1.07	4.19 ± 0.97	0.604
Lymphocytes (10^3^/ml)	2.18 ± 0.72	2.25 ± 0.95	0.609
N:L ratio	2.10 ± 0.70	2.07 ± 0.76	0.745
Postoperative 1st day			
Total leucocytes (10^3^/ml)	13.11 ± 3.94*	12.67 ± 4.60*	0.464
Neutrophils (10^3^/ml)	11.46 ± 3.54*	9.93 ± 4.14*	0.078
Lymphocytes (10^3^/ml)	0.84 ± 0.92*	1.12 ± 0.73*	< 0.001**
N:L ratio	20.73 ± 13.85*	10.19 ± 4.55*	< 0.001**
Postoperative 5th day			
Total leucocytes (10^3^/ml)	12.23 ± 4.75*	9.38 ± 2.23*	0.002**
Neutrophils (10^3^/ml)	9.27 ± 4.78*	5.97 ± 1.86*	< 0.001**
Lymphocytes (10^3^/ml)	1.73 ± 0.64*	1.76 ± 0.71*	0.959
N:L ratio	6.15 ± 3.86*	3.71 ± 1.31*	0.006

Values are mean (± standard deviation) or median (range) as appropriate. *p < 0.05 compared with pre-operative values within the groups. **p < 0.05 compared between groups. Group I: group with cardiopumonary bypass, Group II: group with off-pump coronary artery bypass, N:L: neutrophil:lymphocyte ratio.

Regarding intergroup comparison of values, on the first post-operative day, there was a statistically significant difference between the two groups in terms of absolute lymphocyte counts (0.84 ± 0.92 in group I and 1.12 ± 0.73 in group II, *p* < 0.001). The N:L ratio was also significantly different between the two groups (20.73 ± 13.85 in group I and 10.19 ± 4.55 in group II, *p* < 0.001).

On the fifth postoperative day, there were no statistically significant differences in absolute numbers of lymphocytes or N:L ratios between the two groups. There were however statistically significant differences between the two groups in terms of absolute numbers of total leukocytes (12.23 ± 4.75 in group I and 9.38 ± 2.23 in group II, *p* = 0.002) and neutrophils (9.27 ± 4.78 in group I and 5.97 ± 1.86 in group II, *p* < 0.001) (Table 2).

Regarding the change in total leukocyte, neutrophil and lymphocyte counts, and N:L ratios on the first and fifth postoperative days, from baseline values before the operation, we observed significant intergroup differences on the first postoperative day only (Table 3). There was a significant decrease in lymphocyte counts in group I (52.70 ± 70.83) compared to group II (49.40 ±18.79) (*p* = 0.003), and a larger increase in N:L ratios in group I (1031.78 ± 950.53) compared to group II (472.15 ± 415.93) (*p* = 0.001). The change in other values (total leukocyte and neutrophil counts) were similar in the two groups on the first postoperative day. The change in all counts on the fifth postoperative day was not significantly different in the two groups (Table 3).

**Table 3:** Change in values on the first and fifth postoperative days compared with pre-operative values in the two groups

	*Group I (n = 30)*	*Group II (n = 30)*	*p-value*
Postoperative 1st day			
Total leucocytes (10^3^/ml)	(83.49 ± 74.82)↑	(77.70 ± 42.09)↑	0.802
Neutrophils (10^3^/ml)	(191.16 ± 123.34)↑	(142.43 ± 89.60)↑	0.133
Lymphocytes (10^3^/ml)	(52.70 ± 70.83)↓	(49.40 ± 18.79)↓	0.003*
N:L ratio	(1031.78 ± 950.53)↑	(472.15 ± 415.93)↑	0.001*
Postoperative 5th day			
Total leucocytes (10^3^/ml)	(70.86 ± 79.84)↑	(39.14 ± 45.56)↑	0.154
Neutrophils (10^3^/ml)	(138.00 ± 140.63)↑	(48,91 ± 55.02)↑	0.005*
Lymphocytes (10^3^/ml)	(11.86 ± 46.51)↓	(10.63 ± 42.38)↓	0.442
N:L ratio	(217.94 ± 207.83)↑	(102.74 ± 110.49)↑	0.013*

Values are mean percent (± standard deviation) or median percent (range) as appropriate. *p < 0.05 compared between groups. Group I: group with cardiopumonary bypass, group II: group with off-pump coronary artery bypass, N:L: neutrophil:lymphocyte, ↑: increase, ↓: decrease from pre-operative values.

## Discussion

Surgical stress, regardless of the type of surgery, suppresses cellular immunity as a result of the host’s inflammatory responses. This suppression may be deleterious to the host’s defense mechanisms, along with overproduction of inflammatory mediators.^11^ Some morbidity parameters such as postoperative infection could be predicted from a significant increase in neutrophil and a decrease in lymphocyte counts.^12^

In many previous studies, morbidity and mortality of patients with cardiovascular disease have been demonstrated to be associated with systemic changes in leukocyte subtypes, such as neutrophilia and lymphopaenia, and increased N:L ratios.^13^ In the postoperative period, increased N:L ratio not only assesses the immune condition of the patient but also provides valuable clues regarding morbidity and mortality. In addition, in clinical practice its measurement is inexpensive and simple.^14^

In our study patients, some morbidity markers such as postoperative inotropic need [12 (40.0%) vs 11 (36.7%) *p* = 0.791], postoperative IABCP need [6 (20.0%) vs 1 (3.3%) *p* = 0.103] and postoperative myocardial infarction (MI) [2 (6.7%) vs 0 (0.0%) *p* = 0.492] were found more widely in the CPB group than in the OPCAB group. This is compatable with the literature. Hospital stay was also significantly longer in the CPB group (8.97 ± 2.39 vs 5.90 ± 2.07 days, *p* < 0.001).

Takahashi *et al.* demonstrated that lymphopenia represents an immunodepression status leading to the development of postoperative infection.^12^ It was suggested that lymphopenia may be caused by redistribution between peripheral blood and bone marrow pools in addition to tissue sequestration of activated lymphocyte subsets.^15^

In another study designed by Yamanaka *et al.*, it was reported that the immune system may have been inhibited by neutrophils. Natural killer cells and lymphocytes may have been supressed, and T cells could have been activated by neutrophils in the co-culture of neutrophils and lymphocytes of normal healthy donors. The number of neutrophils added directly affected the degree of supression.^13^

Although conventional CABG surgery with the use of CPB is a safe and effective procedure, it is known to evoke many side effects. It is unique because synthetic non-endothelial surfaces, in which blood continuously recirculates, contribute to the inflammatory response through ‘contact activation’ of the immune system. Aortic cross-clamping causing ischaemia– reperfusion injury to vital organs such as the kidney, brain, myocardium and intestine plays a key role in the activation of a stress–response cascade.^16^

Multi-organ system dysfunction, such as respiratory failure, coagulopathy, renal insufficiency, neurocognitive defects and myocardial dysfunction, occur due to the hyperinflammatory cascades.^17^ Because of the cross relationship between multiorgan dysfunction and the hyperinflammatory state, in our study, patients with postoperative complications such as renal insufficiency, postoperative infection and respiratory problems were excluded from the study population.

Aggregation of WBCs in the capillaries of the lung and degradation of complement proteins, resulting in an activated inflammatory process, cause severe pulmonary dysfunction.^18^ The hyperinflammatory state caused by CPB may play a key role in the genesis of catastrophic complications, leading to postperfusion syndrome, which involves decreased systemic vascular resistance, fever and accumulation of fluid in the interstitial space.^19^ In the literature, there are many studies related to attenuating negative outcomes of CPB by inhibiting the inflammatory response during coronary surgery. Leukocyte depletion,^20^ aprotinin, corticosteroids21 and heparin-coated circuits^22^ are noted as tools to attenuate the inflammatory reactions mediated by CPB.

OPCAB techniques working on the beating heart without extracorporeal circulation may be a more radical and effective way of counteracting the effects of the inflammatory reaction and oxidative stress. Despite great excitement among some cardiac surgeons and patients, the real impact of OPCAB in attenuation of systemic inflammation is still uncertain.^23^ Many clinical comparative studies24 and meta-analyses^25^ have demonstrated shortened length of hospital stay, reduced neurological complications, and reduced hospital costs for patients operated on with OPCAB techniques compared to those undergoing conventional CABG with CPB.^26^ Decreased postoperative blood loss and need for transfusion, and shorter ventilatory support and intensive care unit time have also been reported with OPCAB operations.^27^

Our study had similar follow-up results, showing increased total leukocyte and neutrophil counts and N:L ratios, and decreased lymphocyte counts in both groups of patients, but this was more significant in the CPB group, indicating a more intense inflammatory reaction. In our study, hospital length of stay was longer in the CPB group than in the OPCAB group, which is compatible with the literature.

The inflammatory response due to cardiac surgery is mainly related to the cellular immune system.^28^ During CPB, because of haemodilution, leukocyte counts decrease, but after surgery they increase dramatically.^29^ The contact and complement systems producing kallikrein and C5a strongly activate neutrophils during cardiopulmonary bypass.^30^ IL-6 and IL-8 mediating CPB may partially inhibit apoptosis of neutrophils, and thereby the period of neutrophil activity is prolonged.^31^

Suppression of cellular immunity by the lymphocytes and activation of the inflammatory response, characterised by neutrophilia, are substituted by the N:L ratio. The N:L ratio is increased when lymphopenia or neutrophilia develops. The favourable pattern of changes in systemic leukocyte counts could be defined as a lesser impairment of cellular immunity, determined by lymphocyte counts, and lesser activation of the inflammatory response, measured by neutrophil counts. A favourable pattern of changes in systemic leukocyte counts is indicated by a lesser value of the N:L ratio.^11^

CABG surgery without CPB has been presented as an alternative to minimise the deleterious effects of CPB. The superiority of OPCAB is mostly seen in the clinical era,^32^ but when discussing new techniques, it is also important to clarify the pathophysiology of the procedure. The risk of infection after cardiac surgery is increased with CPB because of neutrophil activation via the complement cascade,^33^ and also because of attenuation of lymphocyte activation.^34^ From the results of our study, we could conclude that a more favourable pattern of N:L ratio was ensured in the early stages of the postoperative period (on the first postoperative day of our study) but later (on the fifth postoperative day), the advantage of the OPCAB technique disappeared, determined by the difference between N:L ratios of the two groups.

From the results of our study, we could infer that the change in N:L ratios on the first postoperative day, compared with pre-operative values, was more dependent on lymphocyte count changes, being more remarkable in the CPB group. On the fifth postoperative day, the change in all values were similar in both groups, compared with pre-operative values.

Although OPCAB has been widely used, no studies have been performed focusing on its effect on the N:L ratio in comparison with CPB. In our study, the groups were similar in terms of pre-operative total leukocyte, neutrophil and lymphocyte counts, and N:L ratios. When compared with pre-operative values, the results indicated that CPB caused significantly decreased lymphocyte counts and increased N:L ratios on the first postoperative day. On the other hand, on the fifth postoperative day, total leukocyte and neutrophil counts were significantly increased in the CPB group.

From these results, the influence of CPB versus OPCAB on changes in leukocyte counts after surgery was shown to be more prominent in the early stages after surgery. In the CPB group, the increase in N:L ratio was more pronounced than that in the OPCAB group only on the first day after surgery. Although we showed that pre-operative values were significantly different from those measured on the first day after surgery, the effect was transient. However, as the magnitude of the response of peripheral leukocytes has been suggested to be related to cardiac surgery, it could be postulated that this transient effect may be greater when the patient undergoes CABG with CPB.

A limitation of this study was that C-reactive protein tests, being an indicator of inflammation, were not done.

## Conclusion

Our data showed that OPCAB compared with CPB could favourably modify leukocyte count changes, including N:L ratio in the peripheral blood stream during the postoperative period of CABG surgery.
